# Redefining the PF06864 Pfam Family Based on *Burkholderia pseudomallei* PilO2_Bp_ S-SAD Crystal Structure

**DOI:** 10.1371/journal.pone.0094981

**Published:** 2014-04-11

**Authors:** Patricia Lassaux, Oscar Conchillo-Solé, Babu A. Manjasetty, Daniel Yero, Lucia Perletti, Hassan Belrhali, Xavier Daura, Louise J. Gourlay, Martino Bolognesi

**Affiliations:** 1 Department of Biosciences, University of Milan, Milan, Italy; 2 Institute of Biotechnology and Biomedicine, Universitat Autònoma de Barcelona, Bellaterra, Spain; 3 European Molecular Biology Laboratory, Grenoble Outstation, Grenoble, France; 4 Unit for Virus Host-Cell Interactions, Université Grenoble Alpes- European Molecular Biology Laboratory-Centre National de la Recherche Scientifique, Grenoble, France; 5 Catalan Institution for Research and Advanced Studies, Barcelona, Spain; 6 Interdisciplinary Centre for Nanostructured Materials and Interfaces and Consiglio Nazionale delle Ricerche, Institute of Biophysics, c/o University of Milan, Milan, Italy; NCI-Frederick, United States of America

## Abstract

Type IV pili are surface-exposed filaments and bacterial virulence factors, represented by the Tfpa and Tfpb types, which assemble *via* specific machineries. The Tfpb group is further divided into seven variants, linked to heterogeneity in the assembly machineries. Here we focus on PilO2_Bp_, a protein component of the Tfpb R64 thin pilus variant assembly machinery from the pathogen *Burkholderia pseudomallei*. PilO2_Bp_ belongs to the PF06864 Pfam family, for which an improved definition is presented based on newly derived Hidden Markov Model (HMM) profiles. The 3D structure of the N-terminal domain of PilO2_Bp_ (N-PilO2_Bp_), here reported, is the first structural representative of the PF06864 family. N-PilO2_Bp_ presents an actin-like ATPase fold that is shown to be present in BfpC, a different variant assembly protein; the new HMM profiles classify BfpC as a PF06864 member. Our results provide structural insight into the PF06864 family and on the Type IV pili assembly machinery.

## Introduction

Gram-negative bacteria virulence is linked to the production of different factors, including Type III secretion systems, flagella, capsule, lipopolysaccharide and Type IV pili (Tfp) [1]. Tfp are the prevalent members of the pili family, building hair-like appendages found on the surface of many bacteria. They participate in adhesion, cell-to-cell interactions, auto-aggregation, biofilm development, DNA exchange and motility [2]. Tfp consist of oligomerized pilin subunits assembled by a Tfp assembly machinery, which shares a common ancestor with the Type II secretion system. Tfp are divided into two types, Type IVa (Tfpa) and Type IVb (Tfpb), based on signal peptide length and the size of their pilin subunits [3]. The study of Tfpb assembly machinery has been pursued to a lesser extent than the Tfpa assembly machinery, since it was held to be very similar to the latter; such a belief was disproved by the identification of Tfpb assembly machinery variants [4], [5]. To date, seven different variants of Tfpb assembly machineries have been identified in Gram-negative bacteria: *i)* the Bundle-forming pilus (BFP) from enteropathogenic *Escherichia coli* [6]; *ii)* the R64 thin pilus [5], [7] of enteropathogenic *E. coli*, together with other R64-related pili from *Pseudomonas aeruginosa* [8] and *Salmonella enterica* [9]; *iii)* the longus (lng) pilus from enterotoxigenic *E. coli* [10]; *iv)* the Cof or CFA/III pilus of *E. coli* [11]; *v)* the toxin co-regulated pilus (TCP) of *Vibrio cholerae* [12]; *vi)* the Cpa pilus of *Caulobacter crescentus* [13]; and, *vii)* the fibril-associated protein (Flp) or tight adherence (Tad) pilus of *Aggregatibacter actinomycetemcomitans* [14].

All components of the Tfpb assembly machinery have now been identified; some (the core proteins) are common to all Tfp systems, while others are specific to each machinery. Little is known about the interactions of such specific proteins in the context of pilus biogenesis, and their functions remain unknown. The global architecture of the Tfpb assembly apparatus is characterized by two sub-assemblies present in the outer (OM) and the inner membrane (IM), respectively, spanning the bacterial envelope. Only two 3D-structures of components of the Tfpb-specific assembly apparatus are known, both being putative stabilizers of the IM complex; these are the N-terminal domain of BfpC [15], and the TadZ protein [16].

A previous study by Essex-Lopresti *et al.* [17] identified eight Tfp-associated loci in the genome of *B. pseudomallei* K96243, a pathogenic Gram-negative bacterium responsible for melioidosis, an often fatal infectious disease that is endemic in tropical areas, particularly in Thailand and northern Australia [18]. Among these, the *Tfp7* locus was recognized as a putative operon/regulon containing nine genes coding for proteins BPSS1593 to BPSS1601. *Tfp7* is of particular interest as it encodes for a Tfpb that is closely related to all members of the R64 thin-pilus variant. The operons of this variant share, along with *Tfp7*, genes encoding proteins belonging to the same Pfam [19] families, and particularly a specific protein, BPSS1599, of unknown function.

Pfam analysis of BPSS1599, a predicted accessory Pilus assembly protein 2 (PilO2_Bp_) revealed an inconsistency between the HMM profile of the PF06864 Pfam family and the proposed PilO2_Bp_ sequence alignment. Based on sequence searches, we present three new HMM profiles that allow a full alignment of PilO2_Bp_ with its Pfam family. Moreover, we report the 1.55 Å crystal structure of the N-terminal domain of PilO2_Bp_ (residues 1-192; N-PilO2_Bp_), solved by S-SAD phasing, providing a first glimpse of the 3D structural properties of the PF06864 family, previously lacking a structure representative. We show that N-PilO2_Bp_ consists of two α/β sub-domains separated by a prominent central cleft, typical of actin-like ATPases. Additionally, in line with the description of PilO2_Bp_ as a putative Tfpb assembly machinery protein, N-PilO2_Bp_ is found to be homologous to N-BfpC, another cytoplasmic protein involved in a Tfpb assembly system. Interestingly, BfpC is a specific protein of the Bundle-forming pilus variant, which differs from the R64 thin-pilus variant to which PilO2_Bp_ belongs. Both structures were found to be very similar, despite lack of any significant sequence identity, and despite the fact that BfpC had not been initially recognized as PF06864 member. Our improved HMM profiles and structural data, taken together, update and improve sequence alignments of the PF06864 family, and shed light on protein 3D structures in the Tfpb assembly machinery.

## Materials and Methods

### Cloning, purification and crystallization

The 5′ end of the *BPSS1599* gene (NCBI accession number YP_111607.1), coding for N-PilO2_Bp_, amino acids 1-192, was amplified by PCR from genomic DNA (Prof. Titball's group, University of Exeter, UK) from *B. pseudomallei* strain K96243 using the primers PilO2-F1 (5′-CACCATGAGCGCGCAGGTG-3′) and PilO2-R1 (5′-CTACGACAGACGCCGCTCG-3′) for insertion into the pET151/TOPO vector (Life Technologies). The same protocol was applied to the 3′ end of the *BPSS1599* gene, coding for amino acids 221 to 432. Successful cloning and PCR fidelity were confirmed by sequencing (BMR Genomics Srl., Padova). N-PilO2_Bp_ and C-PilO2_Bp_ domains were expressed as N-terminal His-tag fusion proteins in C41 (DE3) *E. coli* cells in Luria-Bertani broth, inducing with 0.5 mM IPTG at 18°C overnight. Bacterial cells from a 1 L culture were harvested and lysed in Buffer A (300 mM KCl, 5 mM imidazole, 50 mM KH_2_PO_4_ pH 8), containing lysozyme (0.25 mg/ml), DNases (20 µg/ml) and 10 mM MgCl_2_. Following sonication and centrifugation, the protein of interest was purified using an automated purification protocol (BIORAD Profinia system). To this aim, the soluble fraction was loaded onto a 5 ml Bio-Scale Mini Profinity IMAC cartridge, pre-equilibrated with Buffer A. The protein was then eluted using the standard native IMAC protocol available on the Profinia system, with the addition of 20% glycerol to the elution buffer. Pure fractions, as judged by SDS-PAGE, were pooled and concentrated. The His-tag was removed using the AcTEV protease™ (Life Technologies), incubating 10 mg protein with 55 µl of the protease (10 U/µl) overnight, at room temperature with mild agitation, according to manufacturer's instructions. The His-tag and the protease were removed using the same Profinia system and a 5 ml Bio-Scale Mini Profinity IMAC cartridge. The fraction containing cleaved protein was exchanged into 10 mM Tris-HCl, pH 8 and 20% glycerol and concentrated to 8 mg/ml for crystallization trials. N-PilO2_Bp_ crystals containing phosphate were grown in sitting drops at 20°C, in 300 nl droplets containing 50% protein (8 mg/ml) and 50% reservoir solution (1.3 M sodium-potassium phosphate buffer pH 7.8), using an Orxy8 robot (Douglas Instruments). Crystals grown in the absence of phosphate were obtained from 300 nl sitting drops grown at 20°C, containing 50% protein solution (6 mg/ml) and 50% reservoir solution (0.9 M sodium-potassium phosphate buffer pH 7.0). Crystals were cryo-protected in a solution containing the appropriate buffer and 15% glycerol.

### Generation and validation of the HMM profiles for Pfam family PF06864

A sequential strategy was applied to improve the HMM profiles for the PF06864 family. First, the full sequences of the PF06864 RP15 group members, including PilO2_Bp_, were realigned against the original PF06864 HMM profile using the ‘hmmalign’ tool from the HMMR3 package ^20^ (Alignment 3). Residues comprising alignment positions 1 to 170 in every sequence were then extracted from Alignment 3 for independent alignment. Alignment position 171 contains PilO2_Bp_ Ala93, the first residue aligned with the PF06864 profile. The isolated N-terminal sequences were then used as input for multiple alignment with ‘T-Coffee’ [20], run in three modes: default parameters (Alignment 4A), accurate mode using the EBI psi-blast client (Alignment 4B) and accurate mode using the NCBI blastp client (Alignment 4C). Alignments 4A, B and C were merged with Alignment 3 by substitution of the first 170 residue positions in the latter, thus generating three new alignments, with complete sequences, for the PF06864 RP15 group (Alignments 5A–C). These alignments were used to generate three new HMM profiles (Default HMM profile, EBI HMM profile and NCBI HMM profile) with the ‘hmmbuild’ tool of HMMR3. In order to validate the new HMM profiles, the original PF06864 seed sequences were aligned against them, obtaining three new multiple alignments (Alignments 8A–C). Alignments 8A–C were then compared to the PF06864 seed multiple alignment using the T-Coffee ‘profile *vs* profile’ function, resulting in a score of 98 (out of 100) using the Default and NCBI HMM profiles (Alignments 9 A, B and C, respectively) and a score of 97 using the EBI HMM profile (Alignment 9B). As a reference, a score of 99 is obtained when comparing Pfam's PF06864 seed multiple alignment against itself (Alignment 9D). Finally, the alignment of PilO2_Bp_ and of the rest of the PF06864 RP15 group members (Alignments 6A–C) with the new profiles was analyzed. As expected, the new profiles now cover all the sequence, in contrast to Alignment 1, which excludes the first 92 residues.

### X-ray diffraction Data Collection, Structure Determination and Refinement

Successful S-SAD phasing often relies on the presence of additional/unexpected weak anomalous scattering species (a phosphate ion in our case) in addition to the protein Met/Cys sulphur atoms. The method requires a long wavelength for the incident X-ray beam in order to maximize the f “ anomalous contribution of the S atoms. When using long wavelength (lower X-ray energy) for data collection, the harmonic contaminations of the X-rays affect the anomalous signal. Suppression or reduction of higher harmonic contamination in the primary X-ray beam is an essential precondition for success of an S-SAD experiment. All diffraction datasets for N-PilO2_Bp_ (containing phosphate) were collected on the BM14 beam line, at the European Synchrotron Radiation Facility (ESRF, France) using a MAR 225 CCD detector on a single good quality tetragonal crystal (Space group P4_1_2_1_2, unit cell edges *a = b = *56.0 Å; *c = *117.0 Å; α = β = γ = 90.0°). The 1.55 Å resolution data set was collected at beam energy of 12.7 keV, and treated as a native dataset. The sulfur-SAD data sets were collected at 7 keV (λ = 1.7712 Å) by exploiting the goniostat κ geometry (κ = 0°, κ = 35° and κ = 70°), to limit systematic errors associated with X-ray absorption or radiation damage, and to achieve high multiplicity within the collected data. The harmonic contamination from 21 keV (for the 7 keV set) was reduced by offsetting the second crystal of the beam monochromator using the pusher value -0.25. Datasets were integrated with the program HKL2000, and scaled with SCALEPACK [21]. Data collection statistics are shown in Table 1. Attempts at S-SAD phasing were successful when employing the merged κ = 0° and κ = 35° S-SAD datasets, measured to 1.9 Å resolution. The ShelxC/D/E programs embedded in the HKL2MAP application was used to analyze the heavy-atom substructure of the datasets (*Shelx*C), locate the anomalous scatterers (*Shelx*D), and extract phase information (*Shelx*E) [22]. About 100 trial runs of *Shelx*D were performed to find the correct positions of the anomalous scatterers. All four expected S atoms were located along with an additional peak for a phosphor atom. The ‘heavy atoms’ were subjected to 20 cycles of phase refinement in *Shelx*E, and three cycles of model tracing, while extending data resolution, using free lunch algorithm in *Shelx*E. The experimental phases and the linked model were employed for automated model building in Phenix – *AutoBuild* program [23]–[27], which allowed to build a model consisting of 194 residues, with an overall model/map correlation coefficient of 0.884. Inspection of the map confirmed the presence of one phosphate ion, and of two additional residues at the N-terminal (residues belonging to the vector used). Several rounds of manual model building with COOT, and refinement with the program REFMAC5, were carried over [26], [28], [29]. The structure was refined to R_cryst_ = 0.185, R_free_ = 0.223 values, and the quality of the model checked with PROCHECK [30]. The final refinement statistics and quality parameters are shown in Table 2. The diffraction dataset, for PilO2_Bp_ devoid of phosphate, was collected on ID23-1 beam line, at the European Synchrotron Facility (ESRF, France), on a single good quality tetragonal crystal (Space group P4_1_2_1_2, unit cell edges *a = b = *52.7 Å; *c* = 127.0 Å; α = β = γ = 90.0°). The data were integrated with the program iMOSFLM and scaled with SCALA; data collection statistics are reported in Table 1. Phases were obtained using the N-PilO2_Bp_ bound to phosphate structure by molecular replacement. The structure was further completed manually and refined by cycling between COOT and REFMAC5 programs, refined to R_cryst_ = 0.194, R_free_ = 0.250, and the quality of the model checked with PROCHECK. The final refinement statistics and quality parameters are shown in Table 1. The atomic coordinates and structure factors for N-PilO2_Bp_ with phosphate, and without the bound phosphate, were deposited in the RCSB Protein Data Bank under accession codes 4BYZ and 4BZ0, respectively [31].

**Table 1:** Crystallographic data-collection statistics.

X-ray source	ESRF BM14	ESRF BM14	ESRF ID23-1
Data	S-SAD	Native + PO_4_	Native
Wavelength (Å)	1.7712	0.97872	1.06890
Space Group	P4_1_2_1_2	P4_1_2_1_2	P4_1_2_1_2
Cell parameters (Å, °)	a = b = 56.0;	a = b = 56.0;	a = b = 52.73;
	c = 117	c = 117	c = 127
	α = β = γ = 90	α = β = γ = 90	α = β = γ = 90
No. of molecules in an asymmetric unit	1	1	1
Resolution range (Å)	50–1.9 (1.9–1.93)	50–1.55 (1.55–1.58)	42.33–1.76 (1.76–1.86)
Total Reflections	544748	287763	103017
Unique Reflections	18140	27940	16362
Completeness (%)^a^^,^ b	100 (100)	99.9 (100)	88 (99.1)
Redundancy^b^	30.0 (28.8)	10.3 (10.1)	6.3 (5.9)
Mean I/σ (I)^b^	89.1 (2.92)	35.9 (2.95)	16.6 (5.0)
R_merge_(%)^b^^,^ c	6.0 (16.7)	5.7 (68.3)	5.8 (22.1)
**Phasing** d			
ShelxD Data used (Å)	2.7		
Correlation coefficient (CC)^e^ ShelxD CC_all_/CC_weak_	37.83/23.65		
ShelxE - Figure of merit (FOM)^f^	0.709		

a Data completeness treats Bijvoët mates independently.

b Statistics for the highest resolution shells are given in parentheses.

c *R*_merge_ = ∑*_hkl_*∑*_i_*|*I(hkl)_I_* − < *I(hkl)* >|/∑*_hkl_*∑*_i_*< *I(hkl)_i_* >.

d Substructure determination parameters are from ShelxD.

e CC  =  [∑*wE_o_E*_c_∑*w* - ∑*wE_o_*∑*wE_c_*]/{[∑*w*E_o_^2^∑*w -* (∑*w*E_o_)^2^] [∑*w*E_c_^2^∑*w* -(∑*w*E_c_)^2^]}^1/2^,

where *w* is weight. CC_all_/CC_weak_ is the correlation coefficient for all and weak reflections of the best solution.

f FOM, figure of merit  =  | *F*(*hkl*)best|/|*F*(*hkl*)|; **F**(*hkl*)best  =  ∑*P*(α)**F**_hkl_(α)/∑*P*(α).

**Table 2:** Refinement and Ramachandran plot statistics.

	Native + PO_4_	Native
Resolution range (Å)	1.58– 1.55	1.86 - 1.76
Reflections used for refinement (all)	26448	15597
Reflections used for *R*_free_	1438	851
*R*_cryst_(%)^a^	18.6	19.4
*R*_free_ (%)	22.3	25.0
RMSD bond lengths (Å)	0.008	0.009
RMSD bond angles (°)	1.548	1.372
*B*-factors (Å^2^)		
Protein	19.2	29.8
Water	30.5	36.4
Phosphate ion	23.4	
Potassium ions		30.1
Ramachandran Favored region (%)	92.6	94.8
Additional allowed region (%)	7.4	4.6
Generously allowed regions (%)	0.0	0.0
Outliers^b^ (%)	0.0	0.65

a *R*_cryst_  = ∑*_hkl_*||*F*_o_(*hkl*)|−*k*|*F*_c_(*hkl*)||/∑*_hkl_*|*F*_o_(*hkl*)|, where *F*_o_ and *F*_c_ are observed and calculated structure factors.

b The outlier in the native structure corresponds to conformer A of Arg10, present in a flexible loop region of the structure.

## Results

### PilO2_Bp_ is a component of the Tfpb R64 thin pilus variant

PilO2_Bp_ is described as belonging to the PF06864 Pfam family, which consists of several enterobacterial specific PilO proteins; PilO2_Bp_ is annotated as a specific structural part of a Tfpb assembly apparatus. Notably, two PilO Pfam families have been described and documented in a recent review [32]. The first PilO family, PF04350, hosts proteins of the Tfpa assembly machinery (no homologs to members of this family have been detected in *B. pseudomallei* K96243). The second PilO family, PF06864, includes proteins involved in the Tfpb R64 thin pilus variant, such as PilO2_Bp_. To ease nomenclature, in the following we will address the PF06864 family as PilO2, and add the suffix 2 to all components of the Tfpb R64 thin pilus variant (Pil).

**Figure 1:**
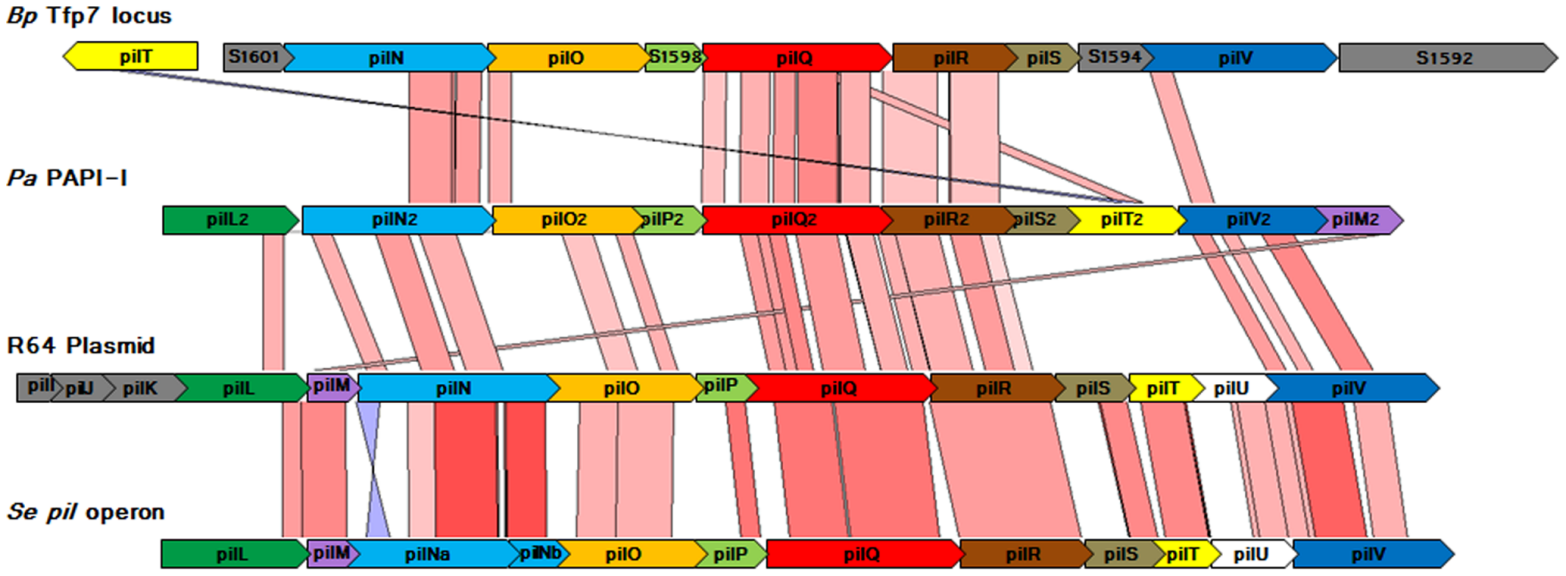
Comparison of Tfpb machinery R64 thin pilus variant encoding operons for different microorganisms. The alignment was performed using tblastx from the Blast suite, and visualized in Artemis Comparison Tool. Conserved protein regions are paired by color-shaded regions; the blue and red colors represent the reverse and forward matches, respectively, and color intensity is proportional to the sequence homology. Genes are represented by arrows; the same arrow color indicates putative orthologs. The grey arrows represent genes lacking homologs among represented *pil* clusters. The *pil* cluster sequences were retrieved from GenBank: *Tfp7* locus from *B. pseudomallei* (*Bp*) chromosome 2 complete sequence, BX571966.1; PAPI-1 *pil* gene cluster from *P. aeruginosa* (*Pa*) PA14, AY273869.1; R64 transfer region, AB027308.1; and *pil* operon from *Salmonella enterica* (*Se*) subsp. *enterica* serovar Paratyphi C strain CN13/87, AY249242.1.

In support of the Pfam family description, we noted that, according to the *Burkholderia* Genome Database [33], PilO2_Bp_ pertains to a predicted operon (positioned from 2167547 to 2177170 bp on chromosome 2 of *B. pseudomallei* K96243), composed of nine open reading frames coding for putative Pil proteins (PilV2, M2, S2, R2, Q2, P2, O2, N2) and for the TcpQ2 protein, with an estimated length of 9623 bp. Downstream to this operon, a *pilT2* gene codes for the PilT2 protein, an additional component of the Tfp7 assembly machinery (Fig. 1). The organization of *Tfp7* is well conserved in all *Burkholderia* strains, suggesting a very ancient origin for this genetic element. Although the genetic organization is not conserved among the Tfpb R64 thin pilus variant operons, all genes are orthologs (Fig. 1). The R64 operon includes 14 genes (*pilI2, J2, K2, L2, M2, N2, O2, P2, Q2, R2, S2, T2, U2, V2*) [5], [7], and was originally described in *S. enterica* serovar Typhimurium; the second operon reported in *S. enterica*, serovar Typhi and serovar Dublin [34], in plasmid pSERB1 from Enteroaggregative *E. coli* [35] and in *Y. pseudotuberculosis* [36] lack the *pilI2, J2, K2 and T2* genes. The organization of the *pil* operon in PAPI-1 from *P. aeruginosa* is somehow different since the *pilM2* gene is at the end of the operon and *pilU2* is absent, the final operon being structured as follows: *pilL2N2O2P2Q2R2S2T2V2M2*. Regarding the Tfp7 operon of *B. pseudomallei*, it is also reorganized as follows: *pilV2M2S2R2Q2P2O2N2,* plus *TcpQ2; pilT2* is found as single gene downstream of the *Tfp7* operon. Thus PilL2 is missing, although it could be replaced by TcpQ2, which has similar function. PilU2 is distantly related to PilD a component of the Tfpa, involved in the processing of prepilin protein PilA, the major subunit pili. As for PAPI-1, *pilU2* is absent from the *Tfp7* operon of *B. pseudomallei*. In the case of R64, *pilU2* encodes for a prepilin peptidase that cleaves PilS2, whereas in *P. aeruginosa* PilD from its Tfpa machinery cleaves PilS2. In *B. pseudomallei*, where six other Tfp's are present, we suggest that one of the prepilin peptidases from a different machinery may process Tfp7 PilS2. The *pilO2* gene is present in all the Tfpb R64 thin pilus variant operons, however its sequence has evolved divergently, leading to reduced sequence homology between *pilO2_Bp_* and the other *pilO2* components.

### PilO2_Bp_ within the PF06864 Pfam family

**Figure 2:**
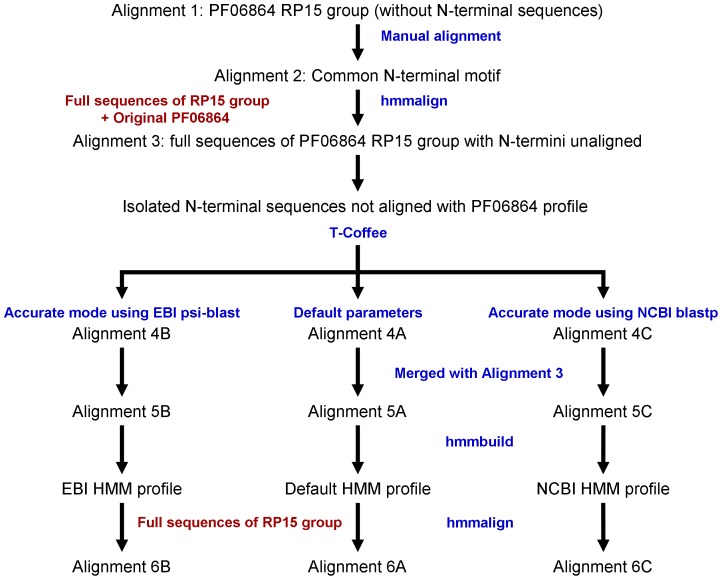
Schematic view of the sequential strategy applied to generate PF06864 Pfam family improved HMM profiles. See the main text and Supplementary Fig. S1. for alignment coding.

As already mentioned, based on the Pfam sequence-search tool PilO2_Bp_ (UniProt [37] code Q63JW5) was assigned to family PF06864. In fact, PilO2_Bp_ is part of the alignment of the proteins from the 15% representative proteomes (RP15) [38] for this family (Alignment 1). However, alignment of PilO2_Bp_ to the PF06864 profile begins at Ala93, thus excluding the first 92 residues and casting doubts on whether PilO2_Bp_ is correctly assigned only as a PilO2 protein. Pfam reports residues 12 to 92 as belonging to the ‘envelope’, however they are out of the alignment and a huge gap is introduced in their place. After careful examination of the PF06864 RP15 it was possible to manually align a short motif in the N-terminal region of these proteins (Alignment 2). This suggested that the first 92 PilO2_Bp_ residues may in fact belong to the PilO2 profile. In order to confirm this hypothesis, a sequential strategy, schematized in Fig. 2, was applied. To see whether the first positions in the family profile may be improved, new HMM profiles were generated. To this end, the program T-Coffee [20] was run in three different modes: default parameters, accurate mode using the EBI psi-blast client, and accurate mode using the NCBI blastp client. The three new HMM profiles and all generated alignments are available for download at http://bioinf.uab.cat/newPF06864hmmprof/. Three complete alignments of the full-length protein, which we shall call Default HMM profile, EBI HMM profile and NCBI HMM profile, respectively, were then obtained and validated for PilO2_Bp_ (Alignments 10 A, B and C). The three profiles produced almost identical results, showing mismatches only in 4 out of the 432 sequence positions. Based on these results, we suggest that the PF06864 Pfam family should be assigned an improved HMM profile.

### Updated HMM profiles identify new members of the PF06864 family

The PF06864 members are described as pilin accessory protein (PAP_PilO) in the Pfam database. The family is composed of 257 protein members, with proteins A3JI41 and Q6EVW5 (UniProt Id) having the lowest and highest scores, respectively. Six of these proteins have been however removed from UniProt, therefore reducing the actual set to 251 members. In search for new members of PF06864, we first scanned the whole uniprot_trembl_bacteria database (http://www.uniprot.org) with the original Pfam HMM profile. Using the hmmsearch program from HMMR3 [39], 391 matches were found with significance above the default threshold, including the 251 proteins reported in Pfam for PF06864 and with A3JI41 as the member with lowest score. All 391 sequences are annotated in UniProt as belonging to the PF06864 family.

We then scanned the uniprot_trembl_bacteria database using our three new HMM profiles. Out of the 251 PF06864 members, 250 scored over the significance threshold. The missing sequence (UniProt Id G1ZDN7) yielded also a positive match when manually aligned against the profiles using the hmmalign program from HMMR3 (204 of the 246 residues in the sequence align using the original Pfam profile, while 184 residues align based on the new Default and NCBI HMM profiles, and 183 with the EBI profile). In addition, and notably, one hmmsearch identified 182 supplementary sequences matching the EBI HMM profile, two of which (G4FYD9 and K2RGC3) have been recently removed from UniProt, and 179 sequences matching the Default and NCBI HMM profiles. The latter 179 sequences are fully contained in the EBI-profile-matching set of 180. Out of the 180 additional proteins, 137 are already annotated in UniProt as members of the PF06864 family. Of the remaining 43 proteins, eight are annotated as PilO or pili-related proteins, two as ATPase, two as BfpC, and 31 as putative or uncharacterized proteins (Table S1). A search in the Swiss-Prot database for proteins matching the three new profiles showed that the majority of matches belong to plant or animal bacterial pathogens, with the remainder being symbionts. Remarkably, similar results were obtained for the 43 newly-identified putative members of the family.

### S-SAD crystal structure analysis of PilO2_Bp_

The function of PilO2 within the Tfpb assembly machinery is not documented, however, PilO2 is reported to be an assembly protein that localizes in the cytoplasm in the absence of other Pil proteins, translocating to the OM in their presence [5]. Given the absence of a signal peptide, the authors suggest that this unexpected intracellular localization is in fact due to complex formation with OM-localized proteins and they propose further investigations into the matter [5]. In order to study the 3D structure of PilO2_Bp_, we first had to define the best strategy to produce the recombinant protein in stable and soluble form. To verify the possible IM localization of PilO2_Bp_, diverse prediction packages were used. SignalP 4.1 [40] predicted the absence of a signal peptide, supporting the hypothesis of cytoplasmic localization. Predicting PilO2_Bp_ topology proved more demanding, due to incoherent results produced by several prediction programs utilized. FFPred, from the PSIPRED website [41], predicted the most plausible topology, with a cytoplasmic localization for the N-terminal domain (1-194), a transmembrane domain (195-214), and a periplasmic localization for the C-terminal domain (215-432). Based on the information emerging from the above predictive approach, an N-terminal domain construct (residues 1-192: N-PilO2_Bp_) was designed. Following expression and purification of the recombinant N-PilO2_Bp_, the 194-residue (the first two N-terminal residues are from the vector) protein was crystallized using the sitting drop vapor diffusion method. The tetragonal crystals, grown from sodium/potassium phosphate solutions at pH 7–8, proved of excellent diffraction quality (see Materials and Methods and Table 1).

Due to the lack of suitable structure homologs in the protein data bank (PDB), the use of molecular replacement to solve N-PilO2_Bp_ 3D structure was prevented. Thus, considering the availability of 1.5 Å resolution data for this protein, the single-wavelength anomalous diffraction (SAD) phasing method was adopted based on the four intrinsic sulphur atoms present in native N-PliO2_Bp_. S-SAD data collection and phasing was conducted at the BM14 diffraction beam line at the ESRF (Grenoble, France), using 1.77 Å X-ray source wavelength. Four sulphur and one phosphor anomalous scatterers were located during the phasing procedure. The N-PilO2_Bp_ 3D structure was then refined using data at 1.55 Å resolution, to R-free and R-factor values of 0.186 and 0.223, respectively (Tables 1–2; for further information see Materials and Methods).

### N-PilO2_Bp_ 3D structure

**Figure 3:**
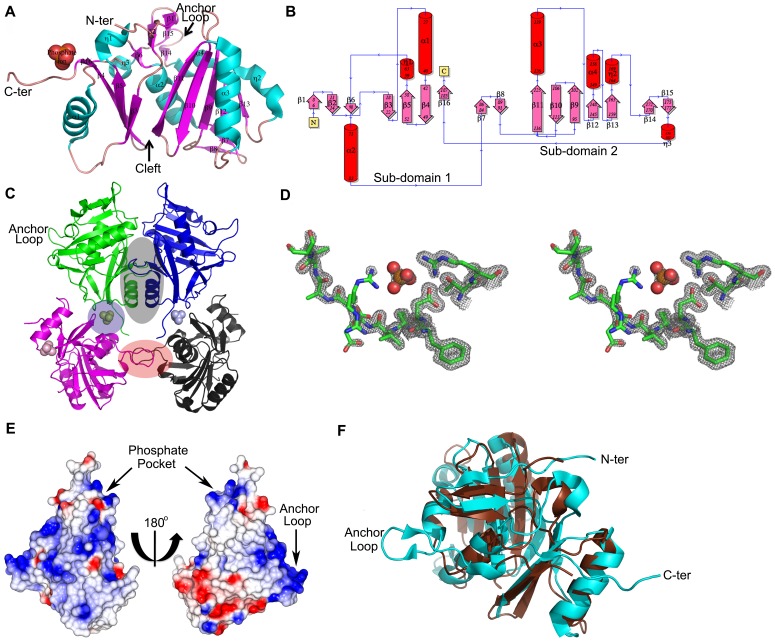
N-PilO2_Bp_ protein. **A.** Overall fold of N-PilO2_Bp_ composed of two α/β topology subdomains, each displaying a mixed β-sheet, separated by a (central) cleft. The bound phosphate ion is shown as spheres. **B.** Topology diagram of N-PilO2_Bp_. This diagram was generated using PDBSum server (www.ebi.ac.uk/pdbsum/) [52]. **C**. Crystal packing of the phosphate-containing N-PilO2_Bp_ structure, showing the three crystal packing dimers formed by alternative interactions between four symmetry-related monomers (green, blue, magenta and black). The three interfaces are highlighted by black, blue and red shading. The first ‘dimer’, is formed by the interaction between the green (or blue) and the magenta (or black) monomers and the light green (or light blue) phosphate. The second crystallographic dimer occurs between the magenta and black monomers. The third dimer is formed by the green and blue monomers. **D**. Stereo view of the electron density map for the residues building the phosphate ion binding pocket. The phosphate ion is shown as sphere; the electron density is contoured at 1.5 sigma level. **E.** Front and back view of N-PilO2_Bp_ electrostatic surface potential. The electrostatic potential was calculated using the CCP4MG viewer. Negative (red) and positive (blue) charges, and uncharged (white) surfaces are shown. **F.** Superposition of the 3D structures of N-PilO2_Bp_ (cyan; PDB codes 4BYZ and 4BZ0) and N-BfpC (chocolate; PDB code 3VHJ).

N-PilO2_Bp_ crystals contain one protein chain (194 residues) per asymmetric unit, structured into two similar sub-domains, each displaying α/β topology, separated by a cleft (Fig. 3A & 3B). This fold, according to the SCOP nomenclature, is typical of proteins belonging to the actin-like ATPase domain superfamily [42]. Indeed, this particular structure results from the duplication of the ribonuclease H-like motif, which consists of three layers (α/β/α), hosting a mixed 5-stranded β-sheet. Such features are conserved in N-PilO2_Bp_ (Fig. 3A & 3B). The protein is divided into two sub-domains. Sub-domain 1 comprises 7 β-strands (β1-6 and β16), one 3_10_ helix and 2 α-helices (α1-α2), comprising mainly N-terminal residues, except for a β-strand 16 formed by C-terminal residues 185-187. As described for the ribonuclease H-like motif, β5 is anti-parallel to β3 and β4 in the sub-domain 1 β-sheet. As for sub-domain 1, sub-domain 2 commences with a β-hairpin (β7-β8) that extends along the lower back of the protein (Fig. 3A & 3B). Subdomain 2 is composed of 7 β-strands (five of which form a β-sheet (β9-β13), 2 α-helices and two 3_10_ helices. From β13, the polypeptide forms an irregular loop that wraps around the back of both sub-domains to finish at the side of sub-domain 1. The two 3_10_ helices are present in this extended loop alongside β14. The cleft at the front of the protein separates sub-domains 1 and 2 (Fig. 3A). The peripheral β-strands (β3 and β11) of the two main β-sheets run antiparallel to each other, and form the walls/floor of the cleft. In full length PilO2_Bp_, Ser192 at the C-terminus of N-PilO2_Bp_ is followed by three residues (Pro-Arg-Ala), and then by the putative transmembrane segment 195-215.

### N-PilO2_Bp_ intermolecular association and biological unit

Although the crystal structure of N-PilO2_Bp_ displays one protein chain per asymmetric unit, inspection of crystal packing highlights three differently packed ‘dimers’ (Fig. 3C). The first ‘dimeric’ interface, results from the interaction of two N-PilO2_Bp_ molecules via a phosphate ion. As explained in the following paragraph, the phosphate ion (that was located through its anomalous scattering signal) could mimic a phospholipid head that may be the true molecular partner recognized by this protein region (res 42-43, 60-61 and 186-192) (Fig. 3D). The second crystal packing dimer is built around an ‘anchor loop’ that covers residues 168-179. This loop was refined with higher than average B-factors, suggesting conformational flexibility that may mediate PilO2_Bp_ interaction/recognition with other assembly machinery partners. In this respect, we found that a native diffraction data set, independently collected on the ESRF beamline ID23-1 from a crystal grown under lower phosphate concentration and at a different pH value, produced an N-PilO2_Bp_ model lacking the mentioned phosphate ion, and presented a larger unit cell (about 10 Å on the *c* edge). Due to even higher flexibility, in the absence of phosphate it was not possible to model the anchor loop structure into continuous electron density. The conformational flexibility of the loop thus appears to be dependent on intermolecular interactions. In fact, in the absence of the phosphate ion the two protein molecules (paired through crystal packing) move apart, in keeping with the increased unit cell size. The third N-PilO2_Bp_ dimer considered presents a wider interface (946 Å^2^) that might be biologically relevant. However, analysis through the PISA server at the European Bioinformatics Institute [43] showed that, despite with a 30-residue interface hosting six hydrogen bonds and six salt bridges, the probability for this homodimerisation interface to be biologically relevant is low (Δ^i^G P-value, 0.303; ΔG, -4.1 kcal/mol; Complexation Significance score (CSS), 0).

The distribution of electrostatic charges shows that two N-PilO2_Bp_ regions are composed of basic residues, with the pocket hosting the phosphate ion and the ‘anchor loop’ (Fig. 3E). Considering the location of the expected transmembrane segment that follows N-PilO2_Bp_ C-terminal residues, such positively charged surface may help the protein interact with a phosphate from IM phospholipid head groups. Indeed, the identified phosphate ion is located in a pocket in the C-terminal region, interacting with Arg61, Arg189 and Asp42. In full-length PliO2_Bp_, such a pocket may face the membrane and fall in its close proximity, thus promoting the interaction with phospholipids. In the crystal, the phosphate ion further interacts with Arg50 and Arg77 of a symmetry-related molecule.

### N-PilO2_Bp_ and the Tfpb bitopic protein N-BfpC share the same fold

As N-PilO2_Bp_ had no evident structural homologs known, we used our crystallographic results to search the structural database. Using Dali [44], the closest structural homolog of N-PilO2_Bp_ was identified as the N-terminal domain of BfpC (Dali Z-score: 16.8, PDB Id 3VHJ, root-mean-square difference (RMSD) of 2.9 Å over 159 matched Cα pairs; hereafter N-BfpC), an accessory protein of the *E. coli* Tfpb BFP variant. As highlighted by the low RMSD value, the two structures are very similar, but differ for the absence of the anchor-loop in N-BfpC (residues 168-179 in N-PilO2_Bp_, Fig. 3F); nevertheless, the two proteins are described as part of two distinct assembly machineries. The next Dali hit corresponds to an uncharacterized protein from *Bacteroides thetaiotaomicron* (PDB 3HRG) with an actin-like ATPase fold (Z-score  = 7.4).

Based on amino-acid sequence only, BfpC is not recognized as a member of a Pfam family, a result that would stress substantial evolutionary distance from PilO2_Bp_. However, a PDB search with the recently introduced PDBfam tool [45] recognizes 3VHJ as the only PDB structure matching the PF06864 Pfam family. Three additional results support BfpC as a member of PF06864. First, alignment of the full BfpC sequence (B7UTD4) against our new profiles resulted in 69% (Default HMM profile), 62% (EBI profile) and 64% (NCBI profile) of the residues aligned. Second, using the function READALIGN of the ProFit program (http://www.bioinf.org.uk/software/profit/index.html) a superposition of the 3VHJ structure onto N-PilO2_Bp_, based on the sequence alignment produced by our HMM profiles produced low RMSD values for the aligned part (3.6 Å using the Default profile, 3.2 Å using the EBI profile and 2.8 Å using the NCBI profile). Thirdly, a search of PilO2_Bp_ against uniprot_trembl_bacteria with the jackhmmer program (HMMR3 package) using default parameters (five iterations) [46] (Pfam main protocol for generating families [19]) returned a multiple alignment where both proteins are present. Using this alignment to superpose both structures results in an RMSD of 3.0 Å. The three pairwise alignments mentioned above, together with a structural alignment obtained with the program CE [47] are presented in Fig. S1.

These findings strongly suggest that both BfpC and PilO2_Bp_, despite their apparent lack of similarity at the sequence level (6.7% identity; 16.7% similarity) (Fig. S2), belong to the PF06864 family. Importantly, these are the first 3D structures assigned to this Pfam family (Fig. 3F).

## Discussion

Tfpb are found in enteric pathogens such as *V. cholerae*, *S. enterica* and *E. coli*, and are important for bacterium-to-bacterium interactions and for pathogenesis [34]. *B. pseudomallei* is a Gram-negative bacterium endowed with high capability of adapting to a wide range of environments, where it can produce biofilm. Both characteristics presumably became possible through the acquisition of genetic information from other organisms. *B. pseudomallei* K96243 hosts eight Tfp machineries, among which Tfp7 is a Tfpb assembly machinery [48]. Such widespread occurrence may imply that Tfp7 is not responsible for the virulent nature of *B. pseudomallei*; however, it may be part of the biofilm formation machinery, as described for the Tfpb R64 thin pilus in *S. enterica*.

We here-report work carried out on PilO2_Bp_, a component of the Tfpb R64 thin pilus variant assembly machinery from *B. pseudomallei*. Despite the fact that PilO2_Bp_, is a representative member of the PF06864 Pfam family, the Pfam algorithm failed to align the first 92 residues of this protein with the other PF06864 members. Pfam is a widely used database of protein families, currently containing more than 13000 manually curated families. Two types of families are distinguished: high quality, manually curated Pfam-A families, and automatically generated Pfam-B families. Some Pfam-A familes are seeded by structures deposited in the Protein Data Bank, and the determination of new structures for known families has already led to their extension in the past [19], [49].

Extensive sequence analysis within the 15% representative PF06864 members indicated that the definition of PF06864 is incomplete. Indeed, the alignment of the N-terminal part of these proteins could be substantially improved using new HMM profiles for the family, as demonstrated here. In addition, screening the uniprot_trembl_bacteria database with our newly developed profiles allowed us to identify 43 new PF06864 family members that are likely to be PilO2 proteins.

Prior to the results reported here, the PF06864 family did not have a representative 3D structure. Our SAD crystallographic approach, based on anomalous scattering from sulfur atoms, yielded a high resolution N-PilO2_Bp_ 3D structure, thus shedding first light on the key structural features of this protein family. The N-PilO2_Bp_ 3D structure, according to SCOP, hosts a ribonuclease H-like fold, typical of proteins belonging to the actin-like ATPase domain superfamily, consisting of a globular moiety composed of two similar α/β sub-domains separated by a cleft.

One of the N-PilO2_Bp_ crystal packing interfaces hosts a phosphate ion, housed in a pocket that could be functionally relevant *in vivo*, mediating the binding to phospholipids of the IM. On the other hand, the stabilization of the (otherwise flexible) ‘anchor loop’ built by residues 169-179 is obtained thanks to intermolecular interactions that occur in a region characterized by positively charged residues, suggesting its potential role in the assembly with other (macro)molecular partners.

N-BfpC is the closest known structural homolog of N-PilO2_Bp_ (RMSD of 2.9 Å). Although BfpC is a Tfpb system component, its assembly machinery pertains to a variant different from Tfp7. However, the two operons share four orthologous genes, with *bfpC* and *pilO2_Bp_* being non-orthologous (sequence identity of 6.7%; sequence similarity of 16.7%). In fact, BfpC had not been assigned to the PF06864 family. Its belonging to this family becomes however clear when the HMM profiles described here are applied. In conclusion, PilO2_Bp_ and BfpC are likely homologous proteins sharing negligible sequence identity but high 3D structural identity (in their N-terminal 194-residue segment), despite the absence of the N-PilO2_Bp_ ‘anchor-loop’ in N-BfpC. Although part of two different machineries, PilO2_Bp_ and BfpC may share similar functions. Such a proposal would be in keeping with the observation that both are accessory proteins in Tfpb assembly machineries, that they comprise two domains linked by a TM helix, and that their N-terminal domains share the same overall fold. Thus, we could speculate that the N-PilO2_Bp_ domain falls in the cytoplasmic compartment, where it might associate with the cytoplasmic domain of the PilQ2 protein (a BfpD homolog) and with the N-terminal domain of the PilR2 protein (a BfpE homolog), in line with the reported association of N-BfpC with BfpD and BfpE [50]. Notably, N-BfpC was identified as a structural homolog of N-EpsL from the Type II secretion system [51]. N-EpsL interacts with the N-terminal part of the EpsE ATPase. The PilT2_Bp_ ATPase from Tfp7 shares 19% homology with EpsE, but lacks the first 110 residues that are responsible for this association. Such structural features may imply that PilT2_Bp_ and PilO2_Bp_ do not associate, or that their mutual recognition is based on different principles.

In conclusion, coupling thorough sequence analyses, database mining, and a new S-SAD phased crystal structure, led to two innovative discoveries within the PF06864 Pfam family. On one hand, the establishment of new HMM profiles enabled a full sequence alignment of PilO2_Bp_ to other members of the family and prompted the identification of 43 new members. On the other hand, crystallographic analysis of N-PilO2_Bp_ provided the first 3D structure of a PF06864 family member, contributing to the characterization of the Tfpb assembly machinery in the R64 thin pilus variant.

## Supporting Information

Figure S1 **Comparison of pairwise sequence alignments of N-PilO2_Bp_.** (Uniprot Id Q63JW5, structure presented in this work) and BfpC (UniProt Id B7UTD4, PDB Id 3VHJ) obtained with different approaches. 1st pair: from comparing Q63JW5 to all sequences in the uniprot_trembl_bacteria database using jackhmmer (HMMR3 package). 2nd pair: Structure superposition using CE. 3rd pair: hmmalign (HMMR3 package) against the Default HMM profile. 4th pair: hmmalign against the EBI HMM profile. 5th pair: hmmalign against the NCBI HMM profile.
(DOCX)

Figure S2 Sequence and secondary structural alignment between PilO2_Bp_ and BfpC.
(DOCX)

Table S1 **New protein assignments to the PF06864 family using to the newly created Default, EBI and NCBI HMM profiles.**
(DOCX)
