# Supplementary material for: Redefining the PF06864 Pfam Family Based on Burkholderia pseudomallei PilO2Bp S-SAD Crystal Structure
Source: PLoS One. 2014 Apr 11;9(4):e94981. doi: 10.1371/journal.pone.0094981 (PMC3984277; doi:10.1371/journal.pone.0094981)
Supplement: Figure S1 — Comparison of pairwise sequence alignments of N-PilO2Bp. (Uniprot Id Q63JW5, structure presented in this work) and BfpC (UniProt Id B7UTD4, PDB Id 3VHJ) obtained with different approaches. 1st pair: from comparing Q63JW5 to all sequences in the uniprot_trembl_bacteria database using jackhmmer (HMMR3 package). 2nd pair: Structure superposition using CE. 3rd pair: hmmalign (HMMR3 package) against the Default HMM profile. 4th pair: hmmalign against the EBI HMM profile. 5th pair: hmmalign against the NCBI HMM profile. (DOCX) [file pone.0094981.s001.docx]

**Figure S1. Comparison of pairwise sequence alignments of N-PilO2_Bp_ (Uniprot Id Q63JW5, structure presented in this work) and BfpC (UniProt Id B7UTD4, PDB Id 3VHJ) obtained with different approaches.** 1^st^ pair: from comparing Q63JW5 to all sequences in the uniprot_trembl_bacteria database using jackhmmer (HMMR3 package). 2^nd^ pair: Structure superposition using CE. 3^rd^ pair: hmmalign (HMMR3 package) against the Default HMM profile. 4^th^ pair: hmmalign against the EBI HMM profile. 5^th^ pair: hmmalign against the NCBI HMM profile.

Legend:

Red: differences in the alignment obtained using jackhmmer relative to the structural one.

orange: differences in the alignment obtained using default hmm relative to the structural one.

blue: differences in the alignment obtained using ebi hmm relative to the structural one.

green: differences in the alignment obtained using ncbi hmm relative to the structural one.

*, $, @, : artificially introduced gaps to keep all correct alignment areas in the same shift.

Q63JW5_jackhammr/1-432 -MSAQVIQIGRQRFVGGLFWQSLSRRNELRAEAVELAKKLKFDLMVLRIDRGV*AAAGYA

B7UTD4_jackhammr/8-401 -----VAVIGSKQYAVNLLWGSSQDTETTNQALNKSLTLMSSKLYSVIGRFQG*EQFAVG

Nt-PilOBpA/1-188 -MSAQVIQIGRQRFVGGLFWQSLSRRNELRAEAVELAKKLKFDLMVLRIDRGV$AAAGYA

3VHJ.pdbA/1-160 KNNLGVAVIGSKQYAVNLLWGSSQDTETTNQALNKSLTLMSSKLYSVIGRFQG$EQFAVG

Q63JW5_defHMM/1-432 .MSAQVIQIGRQRFVGGLFWQSLSRRNE.LRAEAVELAKKLKFDLMVLRIDRGvAAAGYA

B7UTD4_defHMM/10-401 --------IGSKQYAVNLLWGSSQDTETtNQALNKSLTLMSSKLYSVIGRFQG.EQFAVG

Q63JW5_ebiHMM/1-432 .MSAQVIQIGRQRFVGGLFWQSLSRRNEL.RAEAVELAKKLKFDLMVLRIDRgVAAAGYA

B7UTD4_ebiHMM/10-401 --------IGSKQYAVNLLWGSSQDTETTnQALNKSLTLMSSKLYSVIGRFQ.GEQFAVG

Q63JW5_ncbiHMM/1-432 .MSAQVIQIGRQRFVGGLFWQSLSRRNELRAEAVELAKKLKFDLMVLRIDRGv@AAAGYA

B7UTD4_ncbiHMM/7-400 ------AVIGSKQYAVNLLWGSSQDTETTNQALNKSLTLMSSKLYSVIGRFQg@EQFAVG

Q63JW5_jackhammr/1-432 NTRDGFAPGHLSLGAMVSRAIALEGAFYNGRRQPAPNWLGAFALPDGRWAYFAVRD-HAF

B7UTD4_jackhammr/8-401 DKNIGHKRGQVTLLS----AIDFD----------GSSFCGLFPADNELWLVIGVDKDGMV

Nt-PilOBpA/1-188 NTRDGFAPGHLSLGAMVSRAIALEGAFYNGRRQPAPNWLGAFALPDGRWAYFAVRD-HAF

3VHJ.pdbA/1-160 DKNIGHKRGQVTLLSAI---DFDGS-----------SFCGLFPADNELWLVIGVDKDGMV

Q63JW5_defHMM/1-432 NTRDGFAPGHLSLGAMVSraialegafyngrRQPAPNWLGAFALPDGRWAYFAVRDHAFM

B7UTD4_defHMM/10-401 DKNIGHKRGQVTLLSAI-.............DFDGSSFCGLFPADNELWLVIGVDKDGMV

Q63JW5_ebiHMM/1-432 NTRDGFAPGHLSLGAMVSRAialegafyngrrqPAPNWLGAFALPDGRWAYFAVRDHAFM

B7UTD4_ebiHMM/10-401 DKNIGHKRGQVTLLSAI-DF.............DGSSFCGLFPADNELWLVIGVDKDGMV

Q63JW5_ncbiHMM/1-432 NTRDGFAPGHLSLGAMVSRaialegafyngrrQPAPNWLGAFALPDGRWAYFAVRDHAFM

B7UTD4_ncbiHMM/7-400 DKNIGHKRGQVTLLSAI-D.............FDGSSFCGLFPADNELWLVIGVDKDGMV

Q63JW5_jackhammr/1-432 MPNGD-WVGSREEALERLHTDYAWG-GWNVVIGEPELERQGFQNFQPKRLDDLLPRRGGR

B7UTD4_jackhammr/8-401 H--FDKSFHSKDDAKKFFFDHVAYGYPWDRTYSPSDVGVG-----ESRSISELSLI----

Nt-PilOBpA/1-188 MPNGDWVG-SREEALERLHTDYAWGGWNVVIGEPELERQGFQN-FQPKRLDDLLPRRGGR

3VHJ.pdbA/1-160 H--FDKSFHSKDDAKKFFFDHVAYGYPWDRTYSPSDVGV-----GESRSISELSL-----

Q63JW5_defHMM/1-432 PNG-D.WVGSREEALERLHT.DYAWGGWNVVIGEPELERQGFQNFQPKRLDDLLprrgGR

B7UTD4_defHMM/10-401 -HF-DkSFHSKDDAKKFFFDhVAYGYPWDRTYSPSDVGVGESRSISELSLIK--....GK

Q63JW5_ebiHMM/1-432 PNG-D.WVGSREEALERLHT.DYAWGGWNVVIGEPELERQGFQNFQPKRLDDLLprrgGR

B7UTD4_ebiHMM/10-401 -HF-DkSFHSKDDAKKFFFDhVAYGYPWDRTYSPSDVGVGESRSISELSLIK--....GK

Q63JW5_ncbiHMM/1-432 PNGD.WVG@SREEALERLHT.DYAWGGWNVVIGEPELERQGFQNFQPKRLDDLLprrgGR

B7UTD4_ncbiHMM/7-400 -HFDkSFH@SKDDAKKFFFDhVAYGYPWDRTYSPSDVGVGESRSISELSLIK--....GK

Q63JW5_jackhammr/1-432 PRTERWWALRPVERRLSPRAALIAATAACVVLGGAFAYWH-HRAKVEAEEREAALERVRA

B7UTD4_jackhammr/8-401 ----KGKKLKEKGAG-QLMPLLLSGVGIIIFFVIIYNFMNLWLSGRDK--------DILN

Nt-PilOBpA/1-188 PRTERWWALRPVE-----------------------------------------------

3VHJ.pdbA/1-160 ---IKGKKLKEK------------------------------------------------

Q63JW5_defHMM/1-432 PRTERWWALRPVERRLSPRAALIAATAACVvLGGAFAYWHHRAKVEAEEREAALERVRAE

B7UTD4_defHMM/10-401 KLKEKG----AGQLMPLLLSGVGIIIFFVI.IYNFMNLWLSGRDKDILNEEP--------

Q63JW5_ebiHMM/1-432 PRTERWWALRPVERRLSPRAALIAATAACVvLGGAFAYWHHRAKVEAEEREAALERVRAE

B7UTD4_ebiHMM/10-401 KLKEKG----AGQLMPLLLSGVGIIIFFVI.IYNFMNLWLSGRDKDILNEEP--------

Q63JW5_ncbiHMM/1-432 PRTERWWALRPVERRLSPRAALIAATAACVvLGGAFAYWHHRAKVEAEEREAALERVRAE

B7UTD4_ncbiHMM/7-400 KLKEKG----AGQLMPLLLSGVGIIIFFVI.IYNFMNLWLSGRDKDILNEEP--------
