# Supplementary material for: Redefining the PF06864 Pfam Family Based on Burkholderia pseudomallei PilO2Bp S-SAD Crystal Structure
Source: PLoS One. 2014 Apr 11;9(4):e94981. doi: 10.1371/journal.pone.0094981 (PMC3984277; doi:10.1371/journal.pone.0094981)
Supplement: Figure S2 — Sequence and secondary structural alignment between PilO2Bp and BfpC. (DOCX) [file pone.0094981.s002.docx]

**Figure S2. Sequence and secondary structural alignment between PilO2_Bp_ and BfpC.**
