# Supplementary material for: Redefining the PF06864 Pfam Family Based on Burkholderia pseudomallei PilO2Bp S-SAD Crystal Structure
Source: PLoS One. 2014 Apr 11;9(4):e94981. doi: 10.1371/journal.pone.0094981 (PMC3984277; doi:10.1371/journal.pone.0094981)
Supplement: Table S1 — New protein assignments to the PF06864 family using to the newly created Default, EBI and NCBI HMM profiles. (DOCX) [file pone.0094981.s003.docx]

**Table S1: New protein assignments to the PF06864 family using to the newly created Default, EBI and NCBI HMM profiles.**

| **Uniprot Code** | **Score^#^ Default** | **Score^#^ EBI** | **Score^#^ NCBI** | **Protein names** | **Organism** |
| --- | --- | --- | --- | --- | --- |
| **Pilo or Pili related** | |  |  |  |  |
| F8GY55 | 41.8 | 41.8 | 40.5 | ATPase PilO | *Cupriavidus necator* (strain ATCC 43291 / DSM 13513 / N-1) (*Ralstonia eutropha*) |
| Q5NV05 | 37.3 | 36.6 | 36.9 | ATPase (Putative PilO-like pilus assembly protein) | *Ralstonia metallidurans* (strain CH34 / ATCC 43123 / DSM 2839) |
| Q2KY61 | 33.5 | 32.3 | 30.0 | Putative type IV pilus assembly protein | *Bordetella avium* (strain 197N) |
| G2J834 | *BS | 26.0 | 26.3 | Putative PilO-like pilus assembly protein | *Candidatus Glomeribacter gigasporarum* BEG34 |
| D8IZZ0 | 227.3 | 228.3 | 228.0 | Type IV Tfp pilus assembly PilO protein | *Herbaspirillum seropedicae* (strain SmR1) |
| Q5HXR2 | 51.9 | 50.2 | 49.1 | Putative pilin accessory protein | *Gluconobacter oxydans* (strain 621H) (*Gluconobacter suboxydans*) |
| F9RBA5 | 25.8 | 25.7 | 25.8 | Toxin co-regulated pilus biosynthesis protein D | *Vibrio* sp. N418 |
| E9CQX8 | 39.8 | 33.8 | 31.7 | Putative conjugative transfer outer membrane protein PilO (Fragment) | *Serratia symbiotica* str. Tucson |
| **ATPase** | |  |  |  |  |
| L2EDS2 | 40.5 | 38.5 | 38.5 | ATPase | *Cupriavidus* sp. HMR-1 |
| L2EIX4 | 39.4 | 37.4 | 37.3 | ATPase | *Cupriavidus* sp. HMR-1 |
| **BfpC** | |  |  |  |  |
| B3XEB6 | 32.0 | 35.4 | 35.6 | BfpC | *Escherichia coli* 101-1 |
| B1EFR7 | 30.0 | 33.5 | 33.6 | BfpC | *Escherichia albertii* TW07627 |
| **Putative or uncharacterized protein** | | | |  |  |
| F4SY85 | 53.1 | 50.8 | 50.3 | Putative uncharacterized protein | *Escherichia coli* M605 |
| C6BQE8 | 32.1 | 32.1 | 30.8 | Uncharacterized protein | *Ralstonia pickettii* (strain 12D) |
| J7QPH5 | 34.6 | 34.1 | 33.9 | Uncharacterized protein | *Bordetella pertussis* (strain ATCC 9797 / DSM 5571 / NCTC 10739 / 18323) |
| H4WAI2 | 31.0 | 31.3 | 31.5 | Uncharacterized protein | *Escherichia coli* DEC6E |
| D4XAK4 | 25.8 | 27.7 | *BS | Uncharacterized protein | *Achromobacter piechaudii* ATCC 43553 |
| E0WV82 | 108.4 | 104.0 | 104.5 | Putative uncharacterized protein | *Candidatus Regiella insecticola* LSR1 |
| K0MWH2 | 31.7 | 31.3 | 31.7 | Uncharacterized protein | *Bordetella bronchiseptica* (strain MO149) |
| F4GVD7 | 46.5 | 46.4 | 46.1 | Uncharacterized protein | *Pusillimonas* sp. (strain T7-7) |
| E3G6F8 | 28.1 | 28.0 | 28.0 | Uncharacterized protein | *Enterobacter cloacae* (strain SCF1) |
| A4JUJ4 | 30.1 | 28.2 | 27.7 | Uncharacterized protein | *Burkholderia vietnamiensis* (strain G4 / LMG 22486) (Burkholderia cepacia (strain R1808)) |
| H0FA48 | *BS | 26.6 | *BS | Uncharacterized protein | *Achromobacter arsenitoxydans* SY8 |
| D5WNZ8 | 27.6 | 26.8 | 25.5 | Uncharacterized protein | *Burkholderia* sp. (strain CCGE1002) |
| Q7WHL0 | 27.3 | 26.5 | 27.9 | Putative uncharacterized protein | *Bordetella bronchiseptica* (strain ATCC BAA-588 / NCTC 13252 / RB50) (*Alcaligenes bronchisepticus*) |
| I3W449 | 37.7 | 37.9 | 37.8 | Uncharacterized protein | *Salmonella enterica* subsp. salamae |
| Q7VXE6 | 34.6 | 34.1 | 33.9 | Putative uncharacterized protein | *Bordetella pertussis* (strain Tohama I / ATCC BAA-589 / NCTC 13251) |
| I5CLD7 | 39.7 | 38.9 | 39.6 | Uncharacterized protein | *Burkholderia terrae* BS001 |
| D4RI30 | 25.3 | 25.5 | 25.4 | Putative uncharacterized protein (Fragment) | *Enterococcus faecium* E1679 |
| B2TGY1 | 27.0 | 28.0 | 28.0 | Uncharacterized protein | *Burkholderia phytofirmans* (strain DSM 17436 / PsJN) |
| K8REI5 | 30.7 | 31.6 | 29.2 | Uncharacterized protein | *Burkholderia* sp. SJ98 |
| D3SGS5 | 41.2 | 39.8 | 41.4 | Putative uncharacterized protein | *Thioalkalivibrio* sp. (strain K90mix) |
| K0MKA7 | 30.6 | 30.4 | 30.5 | Uncharacterized protein | *Bordetella parapertussis* (strain Bpp5) |
| F2LSL3 | 26.2 | 25.5 | 26.3 | Uncharacterized protein | *Burkholderia gladioli* (strain BSR3) |
| A9IK21 | 33.8 | 33.0 | 30.9 | Putative membrane protein | *Bordetella petrii* (strain ATCC BAA-461 / DSM 12804 / CCUG 43448) |
| L3WXQ1 | 53.3 | 49.0 | 51.5 | Uncharacterized protein | *Escherichia coli* KTE171 |
| G2GXE0 | 146.2 | 142.0 | 144.3 | Putative uncharacterized protein | *Candidatus Regiella insecticola* R5.15 |
| H4VQT6 | 31.8 | 32.1 | 32.4 | Uncharacterized protein | *Escherichia coli* DEC6D |
| K4QE31 | 34.0 | 32.2 | 33.5 | Uncharacterized protein | *Bordetella bronchiseptica* 253 |
| Q7W6M8 | 30.5 | 30.3 | 30.9 | Putative uncharacterized protein | *Bordetella parapertussis* (strain 12822 / ATCC BAA-587 / NCTC 13253) |
| A1TQQ3 | 35.0 | 34.6 | 35.2 | Putative uncharacterized protein | *Acidovorax citrulli* (strain AAC00-1) (*Acidovorax avenae* subsp. citrulli) |
| F4LBD4 | 34.6 | 34.1 | 33.9 | Uncharacterized protein | *Bordetella pertussis* (strain CS) |
| K4U1F1 | 31.0 | 30.6 | 30.8 | Uncharacterized protein | *Bordetella bronchiseptica* 1289 |

*BS: Below significance

^#^ hmmsearch scores (http://hmmer.janelia.org)
